# *Toxoplasma gondii* Actively Inhibits Neuronal Function in Chronically Infected Mice

**DOI:** 10.1371/journal.pone.0035516

**Published:** 2012-04-18

**Authors:** Fahad Haroon, Ulrike Händel, Frank Angenstein, Jürgen Goldschmidt, Peter Kreutzmann, Holger Lison, Klaus-Dieter Fischer, Henning Scheich, Wolfram Wetzel, Dirk Schlüter, Eike Budinger

**Affiliations:** 1 Institut für Medizinische Mikrobiologie, Otto-von-Guericke Universität Magdeburg, Magdeburg, Germany; 2 Leibniz-Institut für Neurobiologie, Speziallabor Nicht-Invasive Bildgebung, Magdeburg, Germany; 3 Leibniz-Institut für Neurobiologie, Abteilung Akustik, Lernen und Sprache, Magdeburg, Germany; 4 Institut für Biochemie und Zellbiologie, Otto-von-Guericke Universität Magdeburg, Magdeburg, Germany; 5 Leibniz-Institut für Neurobiologie, Speziallabor Verhaltenspharmakologie, Magdeburg, Germany; University of Georgia, Georgia, United States of America

## Abstract

Upon infection with the obligate intracellular parasite *Toxoplasma gondii*, fast replicating tachyzoites infect a broad spectrum of host cells including neurons. Under the pressure of the immune response, tachyzoites convert into slow-replicating bradyzoites, which persist as cysts in neurons. Currently, it is unclear whether *T. gondii* alters the functional activity of neurons, which may contribute to altered behaviour of *T. gondii*–infected mice and men. In the present study we demonstrate that upon oral infection with *T. gondii* cysts, chronically infected BALB/c mice lost over time their natural fear against cat urine which was paralleled by the persistence of the parasite in brain regions affecting behaviour and odor perception. Detailed immunohistochemistry showed that in infected neurons not only parasitic cysts but also the host cell cytoplasm and some axons stained positive for *Toxoplasma* antigen suggesting that parasitic proteins might directly interfere with neuronal function. In fact, *in vitro* live cell calcium (Ca^2+^) imaging studies revealed that tachyzoites actively manipulated Ca^2+^ signalling upon glutamate stimulation leading either to hyper- or hypo-responsive neurons. Experiments with the endoplasmatic reticulum Ca^2+^ uptake inhibitor thapsigargin indicate that tachyzoites deplete Ca^2+^ stores in the endoplasmatic reticulum. Furthermore *in vivo* studies revealed that the activity-dependent uptake of the potassium analogue thallium was reduced in cyst harbouring neurons indicating their functional impairment. The percentage of non-functional neurons increased over time In conclusion, both bradyzoites and tachyzoites functionally silence infected neurons, which may significantly contribute to the altered behaviour of the host.

## Introduction

*Toxoplasma gondii* is an obligate intracellular protozoal parasite which replicates sexually in the intestine of its specific hosts, i.e. cats and other felidae. The parasite infects a broad spectrum of intermediate hosts including mice and men. Upon oral infection with oocysts or cysts, *T. gondii* transforms into fast replicating tachyzoites in the intestine of intermediate hosts, disseminates throughout the body, and infects numerous organs including the central nervous system (CNS). In the CNS, tachyzoites infect microglia, astrocytes and neurons Under pressure of the immune system, intra-neuronal tachyzoites transform into slow-replicating bradyzoites, which form cysts [1]. Electron microscopic studies demonstrated *T. gondii* cysts within neurons but not in other cell types of the CNS [2]. However, in up to 50% the definite target cell of the cysts could not be identified. It is thought that the cyst wall separates the parasite from the cytoplasm of the host cell, which in combination with the major histocompatibility complex antigen negativity of neurons results in an escape of the parasite from detection by the immune system [3]. Immune evasion of *T. gondii* is very successful and the parasite persists unlimited in the CNS of its host.

Up to 30% of humans are chronically infected with *T. gondii* and it is generally believed that persistence of *T. gondii* is clinically asymptomatic [4]. However, recent studies suggest that *T. gondii* may alter the behaviour of humans or even increase the risk for neurological diseases including headache, epilepsies, and schizophrenia [5]–[8]. Furthermore, several experimental studies have demonstrated that *T. gondii* manipulates the behaviour of rodents. Importantly, it has been reported that toxoplasmosis converts the natural fear of mice against cat urine into attraction, which may greatly facilitate the transmission of the parasite from mice to its specific host, the cat [9]–[11].

At present it is unclear, how the parasite may alter the behaviour of its host. Since *T. gondii* infects and persists in neurons, the parasite may directly modulate neuronal function. To address this question, we performed combined *in vivo* and *in vitro* studies on the influence of bradyzoites and tachyzoites on neuronal function in *T. gondii*-infected BALB/c mice. In fact, *in vitro* experiments, demonstrated that live but not heat-killed tachyzoites actively prevented normal Ca^2+^ responses of glutamate-stimulated neurons. In good agreement, *in vivo* experiments showed that neurons harbouring *T. gondii* cysts became functionally impaired as indicated by a reduction of neuronal activity-dependent thallium uptake, a potassium analogue. In addition, immunohistochemistry demonstrated that not only cysts but also the cytoplasm of many infected neurons stained positive for *Toxoplasma* antigen indicating that bradyzoite antigens are not sequestered in cysts. Collectively, these findings establish that *T. gondii* alters actively neuronal function and can no longer be regarded as a silent passenger of neurons.

## Methods

### Ethics statement

All animal experiments were approved according to German and European legislation by the Landesverwaltungsamt Halle (Sachsen-Anhalt, Germany; approval number 42502-2-975IfN).

### Animals and infections

Six to eight weeks-old female BALB/c and NMRI were obtained from Harlan-Winckelmann (Borchen, Germany) and kept under special-pathogen free conditions in the animal facility of the University of Magdeburg (Germany). For infection, cysts of a type II strain of *T. gondii* (DX strain) were isolated from the brains of chronically infected NMRI mice (>3 months after infection), counted under a light microscope and adjusted to a concentration of 8 cysts/ml in 0.1 M phosphate-buffered saline (PBS). Finally, 500 µl of this suspension was administered orally to BALB/c mice by gavage. For *in vitro* infection of neurons, tachyzoites of the DX strain were grown in human foreskin fibroblast cell line (originally obtained from the American Type Culture Collection, Manassas, Virginia; cell number SCRC-1041) as described before [12]. Heat-killed *Toxoplasma* were prepared by incubation of tachyzoites freshly relesed from lysed fibroblasts at 65°C for 20 min as described previously [13].

### Behavioural experiments

Infected (day 30 and 60 p..i.) and uninfected control mice were tested in an open field arena (1×1 m, divided into 16 quadrants). In all behavioural experiments, mice were only used once, i.e. only before infection, at day 30 or at day 60 p.i. One day before the experiment, mice were familiarised with the field arena for 10 min. At that time, glass containers filled with saline solution were placed in two opposite corners of the field. The next day, one container was filled with two drops of cat urine and the other with rabbit urine. Mice were placed in the centre of the arena and were allowed to explore it freely for 10 min. Movement of mice was monitored with a digital camera and analysed by an automated tracking system (TSE Systems, Germany). It was calculated how much time mice spent in each quadrant, how often mice crossed lines of quadrants (i.e. visits of quadrant) and how far they moved in each quadrant.

### Imunohistochemistry

For immunohistochemistry, mice were narcotized with ketamine/xylazine (10/0.5 mg per 100 g body weight, i.p.) and perfused intracardially with 50 ml of PBS (pH 7.4) followed by 200 ml of 4% paraformaldehyde in PBS. Brains were removed, post-fixed overnight in 4% paraformaldehyde at 4°C, cryoprotected in PBS containing 30% sucrose at 4°C for 2 days, and, thereafter, frozen in 2-methylbutane at −50°C. Serial frontal brain sections (50 µm) were cut on a cryostat (Leica C 3050, Wetzlar, Germany) and collected in 0.1 M PBS. After blocking of unspecific peroxidase and antigene reactions, sections were incubated with rabbit polyclonal anti-*T. gondii* antibody (Biogenex, Duiven, Netherlands) in PBS with 0.1% Triton X100 for 48 h followed by incubation with biotinylated anti-rabbit secondary antibody (Sigma, Munich, Germany) which was visualized using the avidin–biotin–peroxidase method (ABC-kit, Vector Laboratories, Burlingame, CA) with diaminobenzidine as chromogen. Tissue was counterstained with Haematoxylin & Eosin, cresyl violet and methyl green, respectively. The number and locations of (i) cysts, (ii) infected neurons, (iii) infected glia, and (iv) inflammatory areas were determined microscopically in each brain section of complete brain series. Brain areas were assigned according to a mouse stereotaxic atlas [14]. For immunohistochemical staining, cells were fixed with methanol, blocked against unspecific reactions 15 minutes with a blocking solution (45 ml PBS; 5 ml goat serum, 2.5 g sucrose, 1 g BSA, 150 µl Triton-X 100) and labelled with a rabbit polyclonal anti-T. *gondii* (Biogenex) and anti-rabbit antibody (Sigma) against *Toxoplasma* as well as with mouse anti-class III β-tubulin (BABCO Richmond, CA, USA) and anti-mouse antibody (Sigma) to label neurons.

### Cyst count

The total number of *T. gondii* cysts was evaluated on complete *T. gondii*-immunostained brain section series covering all major anatomic regions of the brain at days 30 and 60 p.i. At least eight sagittal sections per mouse were analysed. In addition, brains were isolated from infected mice and dispersed in 2 ml by passing the tissue through needles with regressing diameter. Finally, cysts were counted microscopically in 25 µl of the brain suspension and the total number of cysts per brain was calculated.

### Thallium application and autometallography

Uninfected control mice as well as at day 30 and 60 p.i. infected mice were used for thallium (Tl^+^) experiments. Intravenous Tl^+^ application and staining were performed according to previously published protocols [15], [16]. In brief, catheters were placed into the right external jugular vein of mice and two days after catheterization mice were injected with 200 µl of 0.05% thallium diethyldithiocarbamate in 0.9% NaCl. Immediately thereafter, mice were anaesthesized and transcardially perfused with freshly prepared 0.325% Na_2_S in 100 mM PBS to fix intra-neuronal Tl^+^ by sulfide precipitation, followed by perfusion with 100 mM PBS containing 3% glutaraldehyde and 0.16% Na_2_S. Finally, brains were isolated, cryoprotected in PBS containing 30% sucrose at 4°C for 2 days, and, thereafter, frozen in 2-methylbutane at −50°C and post fixed with glutaraldehyde. Brain sections were cut in 25 µm thick slices and developed for visualization of Tl^+^. Every third section was counterstained with hemalum or Nissl. Tl^+^ staining was studied microscopically and Tl^+^ positive as well as Tl^+^ negative cysts were count in 50 frontal sections (approx. every third section).

### Live cell Ca^2+^ imaging

Cortical cultures from BALB/c mice embryos (E18) were prepared for *in vitro* calcium imaging. Neurons were cultivated in Neurobasal Medium as described by Xie et.al. [17]. Live cell imaging was performed as previously published [18]. Intraracellular Ca^2+^ responses were recorded by live cell imaging using cortical cultures grown on glass coverslips for 10–14 days. The cultures were infected with tachyzoites or heat-killed *Toxoplasma* of the DX strain at an multiplicity of infection of 1 for 24, 48, and 72 hours, respectively. On the day of experiment, coverslips were incubated with 2.5 µM fluo-4 pentaacetoxy-methylester (Invitrogen, Darmstadt, Germany) for 30 min. Thereafter, coverslips were placed in a stainless steel chamber, mounted on a thermostatically controlled stage (37°C) of an inverted confocal fluorescence microscope (AXIOVERT 100M, LSM PASCAL, Zeiss, Jena, Germany) and superfused (1 ml/min) with HEPES-buffer (140 mM NaCl, 5 mM KCl, 2 mM CaCl2, 10 mM glucose, 10 mM HEPES; pH 7.47) for at least 2 min to obtain a baseline. Thereafter, the neurons were stimulated with 50 µM glutamate for 5 seconds. In some experiments, thapsigargin (2 µM; Sigma, Deisenhofen, Germany) was used to stimulate neurons. Fluorescent images (excitation 488 nm, emission >505 nm) were captured sequentially (10 s intervals). Using Zeiss LSM software the fluorescence intensity was quantified as average intensity within a region of interest after the subtraction of the background values.

### Statistics

Graphpad Prism (Graphpad Software, USA) was used to perform statistical analysis. Behaviour data were analysed by Mann-Whitney U-test for inter-group data comparison and Wilcoxon-test for intra-group data comparison. Students t-test was used for all other set of data. All data are shown as mean value ± SEM.

## Results

### Altered behaviour of T. gondii-infected mice in chronic but not acute TE

**Figure 1:**
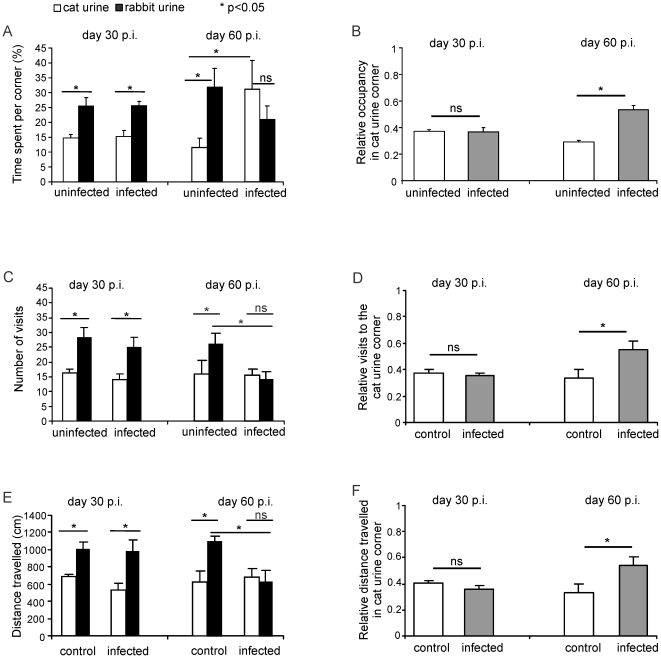
Loss of fear against cat urine in chronically T. gondii-infected mice. (*A*) Compared to non-infected BALB/c mice, no increase in the time spent in cat urine corner was evident in mice at day 30 p.i. (control: n = 6, infected: n = 6). In contrast, at day 60 p.i., infected mice spent a significantly longer time in the cat urine corner as compared to non-infected control mice (p<0.05; control: n = 8, infected: n = 9). (*B*) The relative occupancy of the cat urine corner (ratio of time spent only in the cat urine corner to the total time spent in both cat and rabbit urine corner) was significantly increased at day 60 (p<0.05) but not at day 30 p.i. as compared to non-infected control mice. (*C, E*) Compared to non-infected BALB/c mice, no difference in the number of visits or the distance travelled within the cat and rabbit urine corner were evident at day 30 p.i. (control: n = 6, infected: n = 6). However at day 60 p.i., the number of visits to and the distance travelled within the rabbit urine corner were decreased (p<0.05; control: n = 8, infected n = 9). (*D, F*) The relative visits to the cat urine corner and the relative distance travelled within the cat urine corner were significantly increased in the infected mice at day 60 (p<0.05 for both parameters) but not at day 30 p.i. as compared to non-infected control mice.

It has been previously shown that infection of BALB/c mice with a type II strain (ME49) of *T. gondii* abolished the fear of mice against cat urine. Therefore, we used this model of *Toxoplasma* encephalitis (TE) to further study the interaction of *T. gondii* with the CNS and in particular with neurons. In good agreement with Vyas et al. [11], BALB/c mice infected with the DX strain of *T. gondii* (type II strain) lost their fear against cat urine and spent a significantly longer time in the cat urine corner compared to non-infected mice (Fig. 1A, B). Interestingly, this behavioural change was only observed at day 60 but not at day 30 p.i.. The *T. gondii* induced behavioural changes also included significantly more visits and a longer walking distance in the cat urine corner at day 60 after infection (Fig. 1C–F).

### Amount of cysts varies over time and location

**Figure 2:**
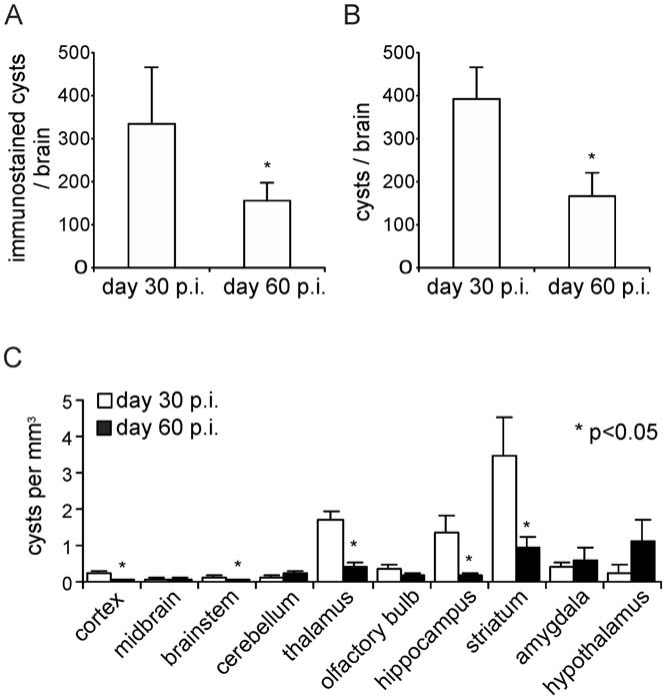
The number of intracerebral T. gondii cysts declines over time in the total brain but not in brain regions important for behaviour. The total number of *T. gondii* cysts was evaluated on complete *T. gondii*-immunostained brain section series at day 30 (n = 4) and 60 p.i. (n = 5). (*A*) The number significantly declined from day 30 to 60 p.i. (p<0.05). (*B*) However, the number of *T. gondii* cysts significantly declined only in the indicated (*) brain regions (p<0.05) but not in the remaining brain regions.

The number of intracerebral *Toxoplasma* cysts significantly declined from day 30 to day 60 p.i. as determined by counting *T. gondii* cysts on brain sections (Fig. 2A) as well as in total brain suspensions (Fig. 2B) indicating that not the absolute number of intracerebral cysts is the decisive factor driving behavioural changes. Interestingly, the decline of cyst number was not observed in all brain regions. A calculation of total cyst number per brain volume of various regions of the brain according to morphometric volume data of Badea et al. [19] revealed that cyst number decreased in cortex, thalamus, hippocampus, and striatum but did not decline or even slightly increased in hypothalamus, amygdala, olfactory bulb, cerebellum, and brain stem at day 60 p.i. (Fig. 2C). Collectively, these data illustrate that the development of behavioural changes was paralleled by the preferential persistence of cysts in defined anatomic structures of the brain.

### Neurons harbour *T. gondii* cysts in different cellular compartments

**Figure 3:**
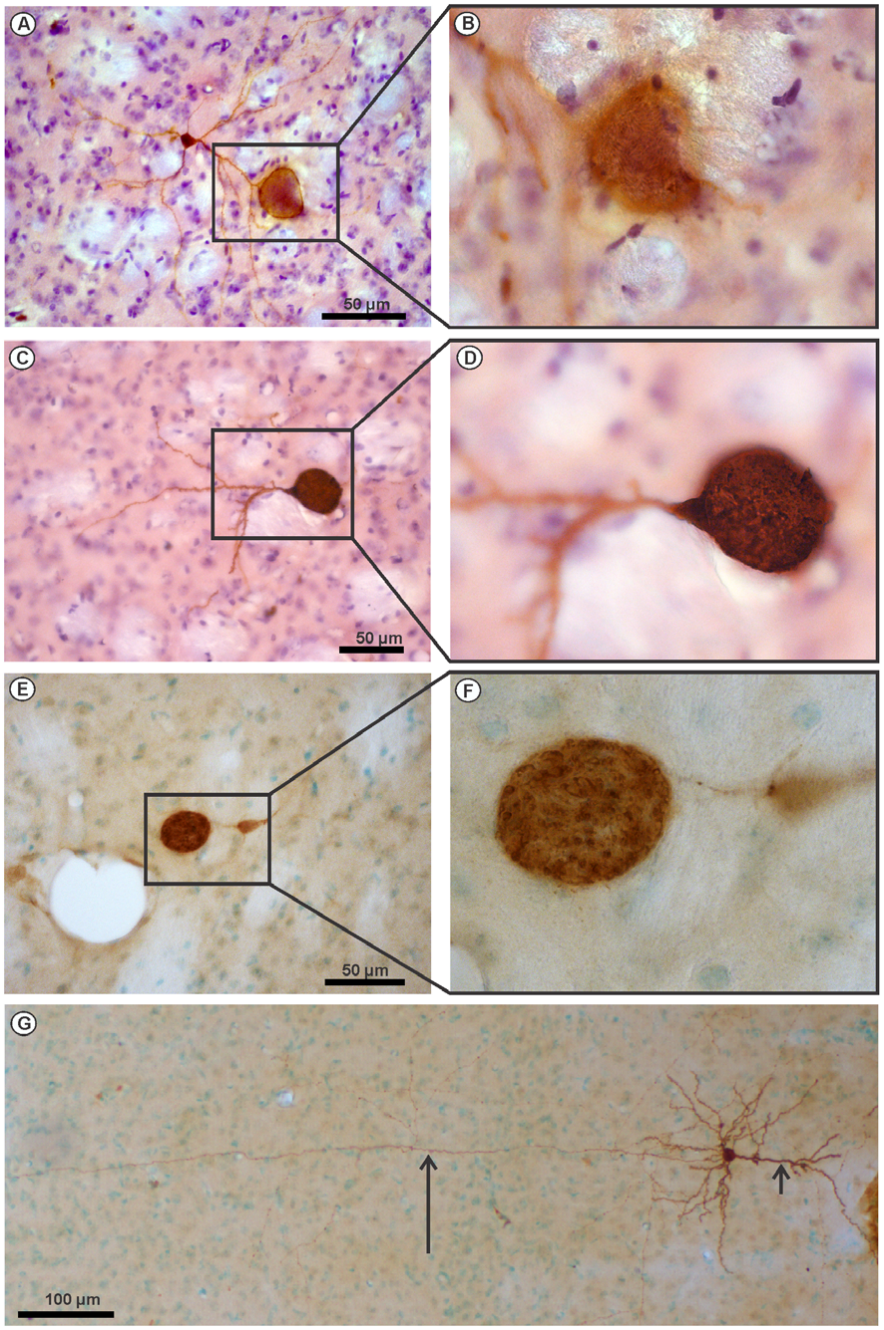
T. gondii cysts reside in all major compartments of neurons. (*A–F*) *T. gondii* cysts were identified in dendrites (*A, B*) (*striatum, day 30 i.p.*), somata (*C, D*) pyramide cell in cortex, day 30 i.p), and axons *(E, F)* (*striatum, day 60* i.p.) in *T. gondii* immunostained brain sections. *T. gondii* antigen was also located outside intraneuronal cysts in dendrites (*A–D, G*), axons (*E–G*), and somata (*A–G*). In (*G*) (cortex, day 60 i.p.), the small arrow points to a dendrite and the large arrow to an axon of a pyramidal neuron which was stained Golgi-like by the *T. gondii* antibody.

So far only Hutchinson and Ferguson [2] have identified by microscopy neurons as the cyst harbouring cell type in the CNS. In good agreement, we also identified neurons as the only cyst harbouring cell population in the CNS. To analyse which parts of neurons are infected by cysts, we performed a detailed immunohistochemical investigation. We observed that cysts resided in all major parts of neurons including the neuronal soma, dendrites and axons (Fig. 3 A–F). This intraneuronal distribution of cysts was detectable both at day 30 and 60 p.i. Importantly, parasitic antigen was also detected outside cysts in a large number of *T. gondii*-infected neurons (Fig. 3A–F) as well as glia cells (not shown). Some neurons were Golgi-like stained by the *T. gondii* antigen and their axons and dendrites could be followed over long distances (Fig. 3 G). Thus, *T. gondii* antigen was not absolutely sequestered in cysts but was also present in the host cell cytoplasm. In neurons, cysts but also *T. gondii* antigens might impact on the function of the host cells to the advantage of the parasite.

### *T. gondii* tachyzoites inhibit Ca^2+^ signalling of neurons

**Figure 4:**
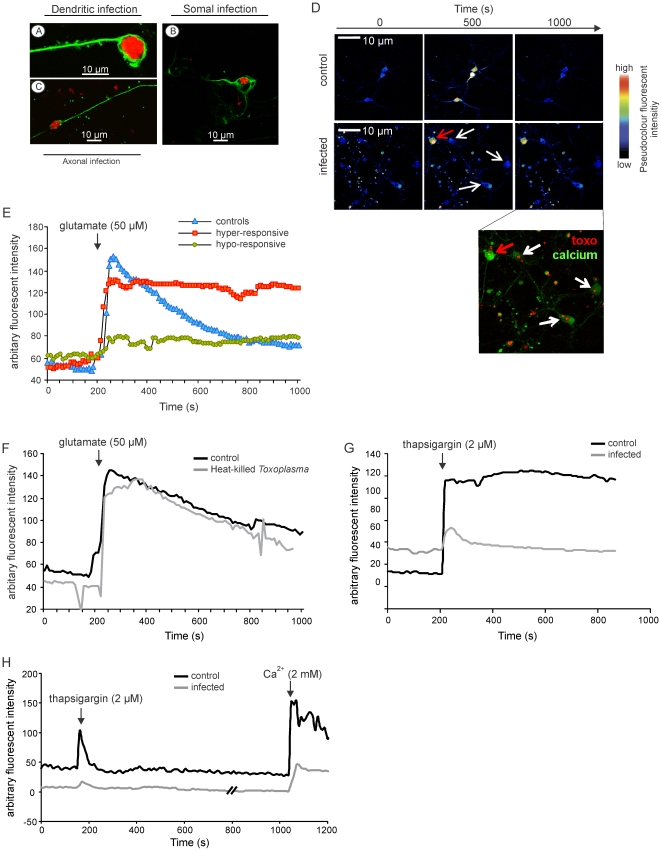
Disturbed Ca^2+^ response in glutamate-stimulated, tachyzoite infected cortical neurons in vitro. (*A–C*) Tachyzoites infect dendrites (*A*), soma (*B*), and axons (*C*) of cultivated cortical neurons as revealed by double immunofluorescence anti-*T. gondii* (red fluorescent) and anti-class III β-tubulin staining (green fluorescent). *(D)* Live cell Ca2^+^ imaging of non-infected control and tachyzoite-infected neurons upon stimulation with glutamate. The time course of Ca2^+^ response is shown as three serial images at 0, 500, and 1000 seconds. Red arrow exhibits a hyper-responsive infected cell while the white arrows demonstrate hypo-responsive infected cells. A counter immunofluorescence staining of the infected culture was done with anti-*T. gondii* showing the presence of toxoplasma tachyzoits in the imaged neurons. *(E)* A graphical representation of calcium responses elicited by controls and infected neurons; control (n = 6), infected hyper-responsive (n = 7), infected hypo-responsive (n = 7). Data are represented as mean ± SEM. (*F*) Neurons infected with heat killed *T. gondii* in-vitro showed no changes in calcium responses upon glutamate stimulation. (*G*) Thapsigargin induces a huge calcium response in the uninfected controls (n = 8) which does not regress due to the inability to reuptake calcium in the endoplasmic stores, but the infected neurons (n = 9) show only a weak signalling upon thapsigargin-stimulation indicating a depletion of intracellular calcium stores.

In order to study the influence of *T. gondii* infections on the neuronal function we performed combined *in vitro* and *in vivo* experiments. Since intraneuronal cysts cannot be induced *in vitro*, we focussed on tachyzoites, which can also infect neurons *in vivo*, and studied the influence of tachyzoites on neuronal function by live cell calcium imaging of tachyzoite-infected murine primary cortical neurons. These experiments revealed that tachyzoites also infected the soma, dendrites, and cytoplasm of the cultivated cortical neurons (Fig. 4A–C). We used an multiplicity of incection of 1, which resulted in infection of approximately 20% of neurons. In non-infected cultures, short-term glutamate stimulation of viable, propidium-iodide-negative neurons resulted in an increase of cytoplasmic Ca^2+^ levels, which dropped after withdrawal of the stimulus (Fig. 4D, E). In addition, non-infected neurons of cultures infected with the parasite showed the same increase of cytoplasmic Ca^2+^ (data not shown). In sharp contrast, tachyzoite infection dysregulated glutamate-induced increase of intracellular Ca^2+^. Approximately 56% of infected neurons were hyper-responsive to glutamate and did not reduce intracellular Ca^2+^ levels after termination of glutamate stimulation (Fig. 4D, E). Remaining infected neurons showed the opposite phenomenon and were non-responsive without showing any increase in intracellular Ca^2+^ levels upon glutamate stimulation (Fig. 4D, E). In contrast to living parasites, heat-killed *T. gondii* did not alter glutamate-induced Ca2^+^ responses in neurons illustrating that active parasitic infection of neurons is a prerequisite for the dysregulation of neuronal function (Fig. 4F).

Thapsigargin, an irreversible SERCA (Sarcoplasmic/endoplasmic reticulum calcium ATPase) inhibitor, induced a sustained calcium response in uninfected control neurons (Fig. 4G). However, in infected neurons, thapsigargin induced either only a short Ca^2+^ response, which dropped within 50 sec to baseline levels, or completely failed to induce any Ca^2+^ increase. These findings indicate that *T. gondii* infection depleted intracellular Ca^2+^ stores, since the major source of Ca^2+^ release induced by thapsigargin is from the endoplasmic reticulum.

### Neurons habouring *T. gondii* cysts become functionally impaired

**Figure 5:**
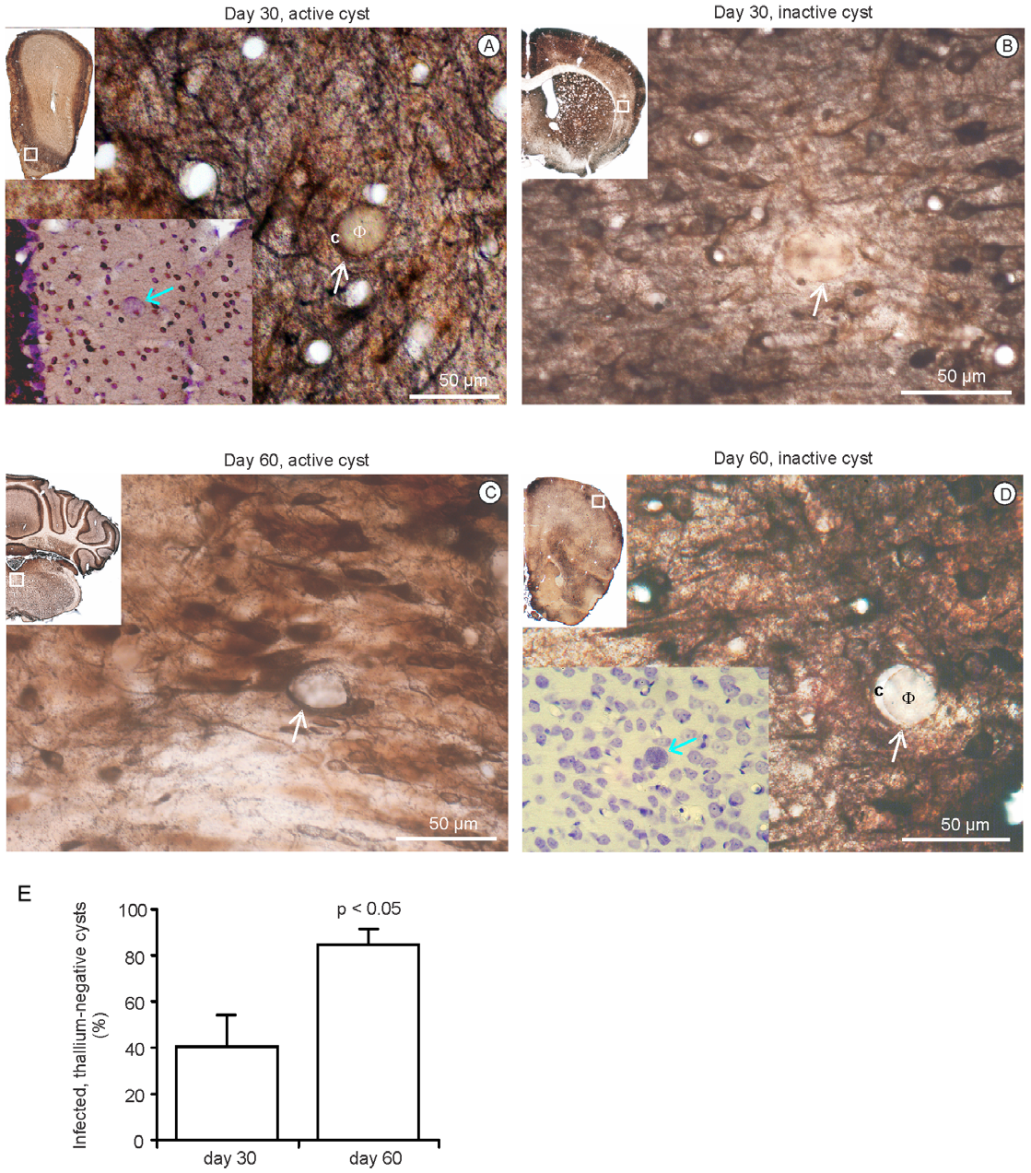
The majority of cyst-infected neurons are functionally silenced in chronic TE. Thallium uptake in *T. gondii* infected mice 30 d p.i. *(A, B)* and 60 d p.i. *(C, D)*. Tl^+^AMG shows T1^+^ positive, functionally active, cyst-infected neurons (white arrows) at day 30 p.i. *(A)* and day 60 p.i. *(C)* as well as Tl^+^ negative cyst-infected neurons at day 30 p.i. *(B)* and 60 p.i. *(D)*. The upper-left insets in *(A–D)* show an overview of the Tl^+^ stained sections and the area (rectangle) containing the illustrated cysts. In *(A, D)*, c depicts the cytoplasm of the infected neurons and Φ the cysts. In *(A)*, lower-left inset, the tissue section has been stained with Tl^+^ and thereafter counterstained with hemalum to show the intact cell wall of the neuron and cyst (blue arrow). Similarly, in *(D)* lower-left inset shows the Nissl-counterstained neuron and cyst (blue arrow). (*E*) A quantitative analysis of Tl^+^ positive and negative neurons was performed at day 30 and 60 p.i. from three mice per experimental group. The total number of cysts as well as the number of Tl^+^ negative cysts per brain were determined on 50 corresponding sections from various brain regions of each mouse. Data show a significant increase of Tl^+^ negative cyst-infected neurons at day 60 compared to day 30 p.i. (p<0.05).

In order to investigate the overall neuronal activity pattern in brains of *T. gondii* infected mice *in vivo* as well as to substantiate the hypothesis of *T. gondii* mediated activity changes at the cellular level (see immunohistochemistry), we analysed neuronal function by thallium autometallography (Tl^+^AMG) experiments [15], [16]. Tl^+^AMG is based on the intimate link between neuronal activity and potassium uptake. Tl^+^ ions are analogous to potassium ions and can therefore be used as a tracer for imaging neuronal activity. Generally, there was no global or regional change of activity in *T. gondii*-infected brains (Fig. 5A–D, overviews). However, at the cellular level our results showed that already 40% of cyst harbouring neurons had a relatively reduced or even no Tl^+^ uptake and, thus, became functionally impaired at day 30 p.i. (Fig. 5B). In chronic TE (day 60 p.i.), the number of Tl^+^ negative cyst harbouring neurons (Fig. 5D) strongly and significantly increased to 78% (p<0.05, Fig. 5E). Importantly, cyst harbouring neurons of all brain regions showed a reduced Tl^+^ uptake at day 60 p.i. suggesting that functional inactivation of neurons in chronic TE is not limited to certain neuronal subtypes. In addition, Tl^+^ negative cysts had normal host cell borders (Fig. 5A, D, insets) illustrating that Tl^+^ negativity was not caused by a rupture of neurons by (reactivated) cysts.

## Discussion

*T. gondii* tachyzoites and bradyzoites can infect neurons and the present study provides for the first time evidence that both stages of the parasite modulate neuronal function. In experiments with tachyzoites, we focussed on the effect of tachyzoite infection on neuronal activity in cultivated primary cortical neurons by live cell calcium imaging, since tachyzoites can not be visualized in Tl^+^AMG stained brain sections. Importantly, tachyzoite infection of neurons resulted in a dysregulated Ca^2+^ influx upon stimulation with glutamate, the major excitatory amino acid in the CNS. In principal, two different Ca^2+^ responses of infected neurons were observed, which both result in functional inactivation of the neuron: either a hyper-responsiveness without terminating Ca^2+^ influx after glutamate withdrawal or a hypo-responsiveness to glutamate stimulation without increase of intracellular Ca^2+^ levels. Since heat-killed parasites did not alter glutamate-induced Ca^2+^ responses in neurons, tachyzoites actively inhibited neuronal function.

The parasitophorous vacuole of *T. gondii*, which is induced upon tachyzoite infection, is in intimate contact with the endoplasmic reticulum of the host cell and interacts with the calcium modulating ligand of the organelle through the parasitic type III trans-membrane protein GRA3 [20], [21]. This interaction might influence the calcium stores in the ER and influence the excitability of the neurons. This assumption is supported by the observation that thapsigargin, an irreversible SERCA inhibitor, induced a sustained calcium response in uninfected neurons, whereas infected neurons responded either with a short Ca^2+^ response, which dropped within 50 sec to baseline levels, or completely failed to increase intracellular Ca^2+^. Both findings in *T. gondii* infected neurons are compatible with a depletion of Ca^2+^ stores in the endoplasmatic reticulum.

Pioneer work of Ferguson and colleagues has identified neurons as the only cyst harbouring cell population in the CNS in 1987 [2]. In extension, we identified that cysts inhibited neuronal activity as revealed by Tl^+^AMG, which enables high resolution mapping of neuronal activity on the single cell level [15], [16]. Neurons that harboured cysts were found to be increasingly unstained by the Tl^+^AMG procedure demonstrating strongly reduced potassium uptake and functional impairment. Importantly, the percentage of Tl^+^ negative cysts increased from 40% at day 30 to 78% at day 60 p.i. Since the absolute numbers of cysts declined from day 30 to 60 p.i. and the number of intracerebral leukocytes in TE peaks at day 30 p.i. and, subsequently declines [13], neither the absolute amount of cysts nor the intensity of the inflammatory reaction but rather the chronicity of infection appears to be important for the altered neuronal function. Interestingly, intraneuronal *T. gondii* cysts become larger over time due to slow multiplication of bradyzoites and, thus, cyst maturation and/or size of the cyst may also functionally impact on neuronal activity. Tl^+^ negative neurons infected with cysts were detectable in all brain regions indicating that the impairment of neuronal function was not restricted to certain neuronal subpopulations.

Immunohistochemistry also revealed that all major parts of neurons including the soma, dendrites, and axons harboured cysts and served as intra-neuronal niches for the persistence of *T. gondii*. Since cysts are much bigger than axons and synapses, the massive, non-physiological enlargement of these neuronal structures may further contribute to a disturbed neuronal function.

In addition to cysts, which were separated from the neuronal cytoplasm, intraneuronal *Toxoplasma* antigen was present in the cytoplasm of cyst harbouring neurons and *T. gondii* antigen positive axons could be followed over long distances. This latter observation demonstrates that cysts and parasitic antigens are not completely sequestered from the neuronal cytoplasm. Thus, similar to tachyzoites, bradyzoites may have the capacity to transport parasitic molecules across the cyst wall into the neuronal cytoplasm. Potentially, this might enable bradyzoites to modulate neuronal function. An effect of secreted, tachyzoite-derived parasitic molecules on host cell function has been demonstrated before by in *vitro studies* [22], [23]. These experiments revealed that tachyzoites release parasitic molecules into the host cell cytoplasm in order to modulate various signalling pathways, in particular apoptotic pathways, in order to guarantee host cell survival, which is crucially important for this obligate intracellular parasite. Our detection of parasitic antigens in the cytoplasm of cyst harbouring neurons, implies that cysts also ensure their intraneuronal survival by the active inhibition of neuronal apoptosis [24]. In fact, this might be critically important for neuronal survival, since cysts inhibited neuronal function and non-functional neurons are prone to apoptosis.

Noteworthy, bradyzoites slowly replicate in cysts, which may result in cyst rupture and release of parasites from neurons. In fact, it is thought that cyst rupture is a common event in the CNS of chronically infected individuals and results in the infection of other neighbouring neurons [25], [26]. Since cyst rupture might affect neuronal activity, we co-stained Tl^+^-labelled sections also for their cytoarchitecture (hemalum or Nissl stain). These experiments revealed that both cyst walls and neuronal cell membranes were intact illustrating that intact but not ruptured cysts inhibited neuronal activity.

The manipulation of neurons by *T. gondii* may not only facilitate the persistence of the parasite in the CNS but also contribute to the efficient transmission of the parasite from an intermediate host to its specific host. In the natural life cycle of *T. gondii*, the transmission of the parasite from mice to cats plays a pivotal role and, therefore, the loss of fear against cat urine odor may be specifically induced by the parasite-mediated manipulation of the CNS and the functional impairment of infected neurons. Importantly, (i) the functional impairment of neurons by cysts, (ii) the high number of cysts in behaviourally relevant brain regions, and (iii) the loss of fear against cat urine odour all increased over time. Since the total number of intracerebral *Toxoplasma* cysts declined significantly from acute to chronic TE, factors other than the total intracerebral parasite load were responsible for the loss of fear against cat urine odour. In this context, it is remarkable that the number of cysts did not decline but slightly increased over time in the hypothalamus and the amygdala. This confirms previous findings by Vyas et al. [9], [11] which also observed a relative high amount of cysts in the amygdala of *T. gondii*-infected BALB/c mice, which led to the suggestion that the presence of *Toxoplasma* cysts in brain areas important for behaviour might be responsible for the behavioural changes. However, in contrast to Vyas et al. we did not observe an attraction of infected mice towards cat urine and additionally could not detect behavioural alterations in acute TE. These differences may be explained by the use of different type II strains as well as different doses of infection in our and the previous study.

It has been reported that *T. gondii*-infected humans suffer significantly more frequently from epilepsy as compared to non-infected persons [5], [7], [27]. In addition, several studies reported an altered behaviour of *T. gondii* infected humans [28], [29]. Although it is tempting to speculate that (i) *T. gondii*–induced hyper-responsiveness to excitatory amino acids, as observed in our in vitro experiments, may contribute to the development of epilepsy and (ii) *T. gondii*-mediated hypo-responsiveness of infected neurons may cause behavioural changes, we would like to emphasize that these human disorders are complex and that it is unlikely that *T. gondii* can induce these diseases without other crucial co-factors. Even if *T. gondii* contributes to the development of these disorders, various parameters of TE in addition to direct manipulation of neurons by the parasite could play a role including inflammation and changes in neurotransmitter production [1], [29], [30].

In conclusion, both tachyzoites and bradyzoites functionally impair murine neurons and, thus, can no longer be regarded as silent passengers persisting in its intra-neuronal niche. An exact identification of the mechanisms how bradyzoites and tachyzoites interfere with neuronal function but still guarantee the survival of the functionally impaired neurons will enable the design of new drugs with the potential to eradicate persisting *Toxoplasma* from the CNS.
